# Texts, Practice and Practitioners: Computational Cultures at Work in Early Modern South India**

**DOI:** 10.1002/bewi.202200012

**Published:** 2022-11-28

**Authors:** D. Senthil Babu

**Affiliations:** ^1^ Institut Français de Pondichéry and ETH Zurich

**Keywords:** mathematical practice, village accountant, South India, *Kaṇakkatikāram*, Tamil, history from below

## Abstract

This essay will discuss the hegemonic role that texts have come to play in the historiography of subcontinental mathematical traditions. It will argue that texts need to be studied as records of practices of people's working lives, grounded in social hierarchies. We will take particular mathematical texts to show how different occupational registers have come to shape practices that defy the binaries of concrete and abstract, high and low mathematics or the pure and applied conundrum. Measuring, counting and accounting practices as part of the routine work of practitioners performing their caste occupations then provide us with a spectrum of the computational activities that controlled and regulated the lives of people in the past. In the process the act of computing itself gained certain political values such as cunning and manipulation, identified with professions of village accountant and merchant, for example. Drawn from my earlier work on these records, I discuss the occupational role of the accountant as a political functionary who assessed and authenticated the measurements of land and produce in the village, making values of the labor performed by others, and creating avenues for his own proficiency as a mathematical practitioner.

## 1 Introduction

Histories normally help us to ask and answer certain basic questions about the past, such as who did what, when and in what context? How did people do what they did and what were the consequences of their actions, to others and to themselves? How did they choose to represent their activity, in what manner, and what purpose did such representations serve, for whom and how? However, such inquiries seem to have bypassed the writing of histories of mathematics in general and more particularly in the case of histories pertaining to mathematics in the Indian subcontinent. This is probably because historians have been preoccupied with writing the prehistories of a particular kind of mathematical activity, which characterized mathematics as a disciplinary field, recognized as a subject of teaching and learning in schools and universities, which in turn is itself only a recent phenomenon in the Indian context, starting from about the mid‐nineteenth century during British colonial rule.^1^ Yet this paper will show how the computing activities of the village accountant's occupation related to the ordinary people in these societies. For this purpose, the paper uses non‐traditional sources which include textual records of practices of these accountants and popular perceptions of the people about them. It then traces the changes in their occupations and their relationship with the society during the British colonial rule.

Traditional histories had a political function, namely to constitute a particular imagination about mathematics. This imagination is characterized by a sense of privilege of the activities of the mind over those of the hand. This could be a universal problem in history but in societies that are socially fragmented as in the Indian case, through ritually sanctioned caste as a social and political institution, the equally universal quest for the dignity of labor then requires the rewriting of histories. Given this background, the question is raised how we can reconstruct histories of mathematics that would help us in the task of democratizing knowledge in socially fragmented contexts. This task defines the program a group of colleagues and I are building through the ongoing project *History of Vernacular Mathematics in Medieval South India (Malayalam and Tamil, 9^th^–16^th^ Centuries)*, a collaboration between the French Institute of Pondicherry and the Chair for the History and Philosophy of Mathematics at ETH Zurich. The project aims to develop a social history of mathematical practices in India.^2^

We understand that writing different histories cannot be a history of disputes over priority, where the preoccupation is with the question of who did what first, which politically assumes that social activities of borrowing, translating, and reshaping knowledge between cultures is an inferior act as compared with “original” creations of knowledge. We value the contributions of history writing attempting to find affinities in thought processes across cultures, by tracing parallels and similar forms of knowledge in context—something that a tradition of post‐war humanist scholarship taught us—where the emphasis was on “tracing mathematical sameness across time and space,”^3^ But still these histories do not help us to understand mathematical activities of the past as social practices, performed by real people in a particular time and space. Our concern is to prevent histories from perpetuating schisms or binaries such as mental and manual, theory and the practical, pure and applied, concrete and abstract. We believe that such binaries aid and facilitate hierarchies in already fragmented societies and could thus hinder attempts at democratizing knowledge. We would rather attempt a different kind of history writing showing how men, women, and children were or were not able to come together to make knowledge part of their routine activities of working and learning. Therefore, we choose to study historical sources such as texts as records of practices of particular practitioners in specific times and spaces.

When such practices and practitioners become the agents for reconstructing histories, interesting avenues open up to access their world. Take for example a poignant and telling moment from third‐century BCE Mesopotamian society, when a scribe was telling his junior to do his job properly and measure correctly so that brothers do not attack each other.^4^ Interestingly this resonates very closely with the voice of Suryabooban, a sixteenth‐century revenue accountant of Guntur district in the present day state of Andhra in South India, who constantly reminded his students of the necessity for computational correctness as a normative virtue that comes out of repeated and hard practice, in such a way that the everyday never becomes routine but is rather a responsible practice.^5^ When mathematical activity becomes work, the artisan, the accountant, the astronomer, the teacher, and the student become computational practitioners serving definite purposes and doing everyday jobs. Such orientation towards work created particular kinds of mathematical practice, which need to be understood only as instances in the political economy of computational work in specific historical contexts.

In this paper, I therefore want to bring to the fore the practitioner and thus write a history from below with respect to the history of mathematics in India. I argue that this in itself is insufficient; what is required is to bring in the public, whom the practitioner through his occupational practice routinely encountered. Practices and public together constitute a field for historical scrutiny in ways do not privilege a particular kind of knowledge activity divested from its consequences for the people; this could be the beginning of a history of mathematics from below in the South Indian context. I shall deal with the computational work of one practitioner: the village revenue accountant, based on the records of occupational practices available as texts and on the cultural perceptions about the occupational work of this functionary, found in Tamil folklore and literature. The village accountant had to confront his public, or the subjects of his occupational practice, who perceived his work predominantly as manipulation and deception, giving rise to an image of the computational mind as a cunning mind, one which manipulates, plots, exploits, and destroys. There is another practitioner in South Indian history, the merchant, who gained a similar reputation with the public.

Both the village accountant and the merchant usually belong to a particular caste. The Chettiars, as a caste are known for their mercantile ventures, their rigorous accounting practices identified with entrepreneurship, and as a community that attempted to play a role in the making of a capitalist trading and finance network across the Bay of Bengal, crossing the oceans into the foreign lands of South‐East Asian countries in the late nineteenth and early twentieth centuries.^6^ But even today, the prevalent cultural perception of the Chettiars among the Tamil public is as a community reputed for their cunning, inciting suspicion, and ridicule at the same time. What the accounting practices of these mercantile communities were and how they were trained in the computational arts still awaits the attention of the historian of mathematics. It is important to point out that the politics of public perception need to be integrated into the study of the practices of the mercantile practitioner of the computational arts.^7^ For the purpose of this essay I shall stick to the accountant by first providing an introduction to the kind of textual sources available, which can be studied as records of the practices of the accountant, and second, by discussing the occupational role of the accountant so as to situate his computational work as political work. I will discuss his work in the precolonial Tamil society and then briefly point to the way in which the nature of his computational work changed during the transition into the rule of the British East India Company, when private property was established bringing in a different order of practice, not only in revenue accounting but also in changing the character of numeracy and the place of numbers in early nineteenth‐century Tamil society.

## 2 Texts and Their Practitioners^8^

First, it is necessary to introduce certain textual sources, which I argue are records of practices in both the school and the work place of the accountant. The first set of texts are number tables called *Eṇcuvaṭi*, which contain elementary arithmetical tables in the Tamil system. These texts as manuscripts are available in various manuscript libraries in South India.^9^ They are considered to be from the eighteenth century or even earlier. The surviving manuscripts in these repositories are only a small number, given that till today in several rural households in different parts of the Tamil‐speaking region, we keep finding these in the households of people. The second set of texts are called *Kaṇakkatikāram* texts, which detail the engagement of the accountant's computational practice as part of his occupation. These texts are considered to be from about the sixteenth‐century and are available in the manuscript libraries in South India.^10^ I shall briefly discuss how the pedagogic practices of the school related to the accountant's practices at work, before I focus our attention on the nature of his work in the agrarian social order.

*Eṇcuvaṭi* texts consist of number tables, tables of land, capacity, weight and time measures, multiplication tables involving whole numbers and fractions, tables of squares, and names of Tamil months and years. There is a strong pedagogic basis to their very organization. These texts are not textbooks in the modern sense but products of learning generated by the arithmetic practices of a specific location, the elementary *pathshala*, or *tiṇṇai* school. These were elementary schools in the precolonial Tamil society,^11^ where every student during the process of memorizing the various arithmetic tables wrote their own *Eṇcuvaṭi*s. The students would begin their learning of arithmetic with the number tables with the practice of reciting aloud and simultaneous writing. The writing of the entire table book on palm leaves by each student marked the culmination of the learning of arithmetic—an end product of their training. I have demonstrated how the precolonial elementary schools sustained the learning of arithmetic through recollective memory‐centered pedagogic practices, of which the *Eṇcuvaṭi* was the product.^12^ The school remained a central agency in imparting training and nurturing skills in the mnemonic computational arts till the late century. The *Eṇcuvaṭi* culture of learning ensured that certain basic functional skills in language and arithmetic were provided to Brahmins and other land owning upper caste sections, but not to the oppressed caste social groups in Tamil society during the eighteenth and nineteenth centuries, if not earlier.^13^ These schools sustained the learning of the *Eṇcuvaṭi* mode of arithmetic while in turn feeding into the learning of professional computational practices, like that of the occupation of the village revenue accountant or of the merchant's accountant. These two processes probably complemented each other while making possible the production of specialized knowledge.

**Figure 1:**
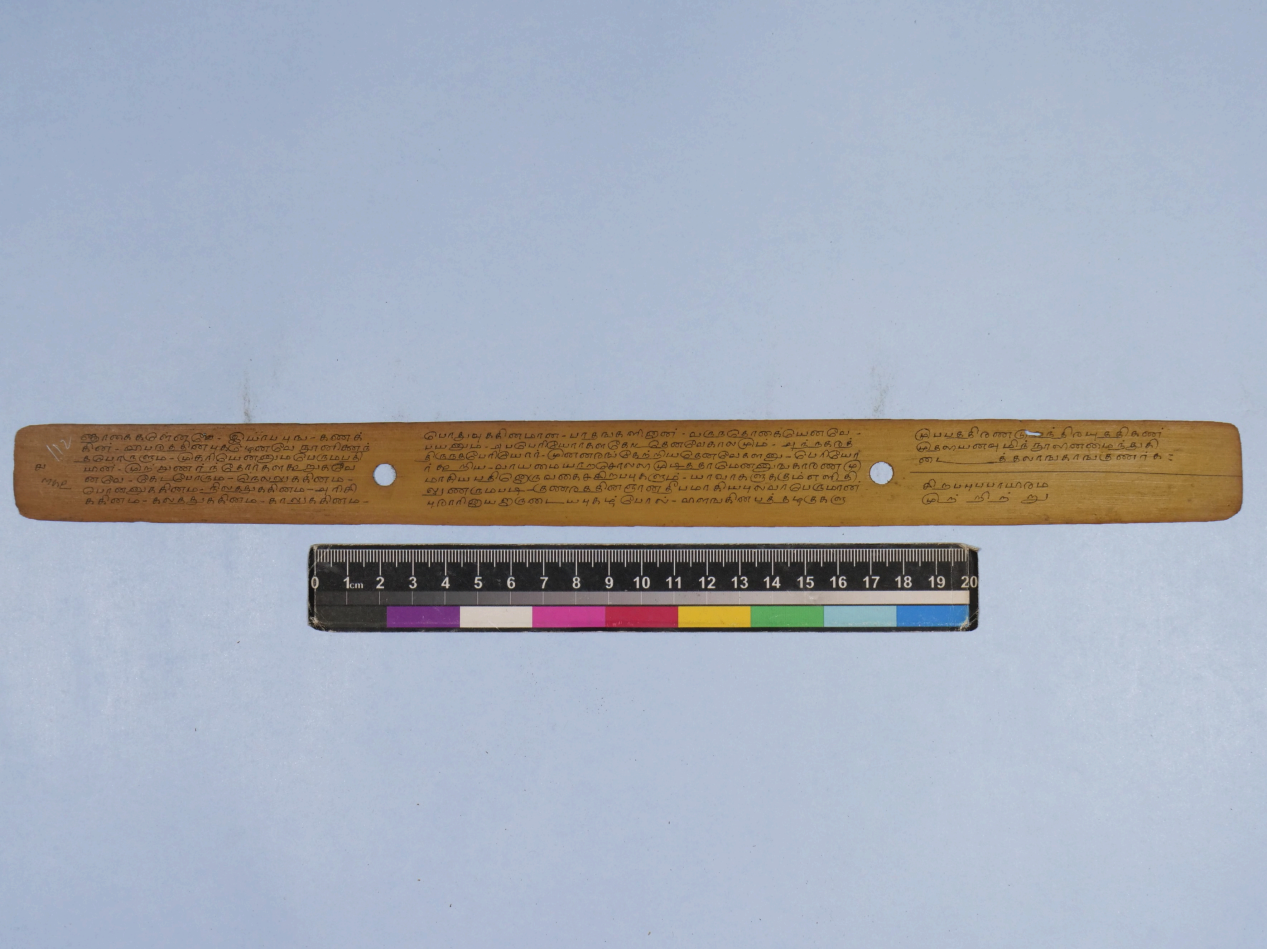
A leaf from the manuscript of *Kaṇakkatikāram* (catalogue no. TR222). Courtesy: Government Oriental Manuscripts Library, Chennai.

The *Eṇcuvaṭi* training mediated the activities of measuring in the realm of production and distribution, when they were calculated through a world of computing them as proportions. These computations often happened away from the sites of production. Such computational contexts tested and honed training in the mnemonic arts imparted in the school through the *Eṇcuvaṭi* so that computations can be performed correctly and swiftly. The student's continuous refinement required more problem types, as the recall of the right procedure at the appropriate instance provided the solution to a given problem, be it in the sphere of field mensuration or that of computing proportions of produce. The table book version of *Eṇcuvaṭi* was an essential resource for this mode of problem solving. The recollection of the appropriate table for the correct number in the context of the arithmetic operation involved made the solution of the problem possible. Even if the quantities involved were of different magnitudes, the training in the number tables enabled the practitioner to convert them into basic units and proceed further. This is why an ideal practitioner associated particular procedures with a problem type, backed with memory tables. Memory, then, was a virtue in this system. Memory combined with prudence in identification of the correct procedure for initiating problem solving constituted a skill. This skill was considered worthy of being nurtured and developed, as if the society had consciously decided to develop a particular expertise in its skilled labor.^14^ The requirements of the job and the training in the school complemented each other. But the work place had to take over the pedagogic task after the school, by shifting the modes of training to the fields of practice, where the people, whose labor and lives were constantly assessed and valued, in this workplace, constantly watched over the practitioner. This is where I situate the *Kaṇakkatikāram* texts, which show us the connection between the *Eṇcuvaṭi* training and the job of the accountant (Figure 1).^15^

The *Kaṇakkatikāram* texts are records of the computational practices which connected the pedagogic and the work place, retaining characteristics from both these spheres. These texts are available as palm‐leaf manuscripts in libraries and also as printed editions.^16^ These texts are organized into different sections, which show that they follow a classification of the computational arena in line with the normative techniques employed to solve problems. These problems are primarily concerning situations when objects need to be measured, counted, and assessed. Measuring land, weighing gold, computing wages, distributing produce, estimating, and computing time constitute the orientation of these texts. I will briefly point out problem situations using the 1783 manuscript of *Kaṇakkatikāram* to demonstrate how different people and their occupations in the context of their computational activity occur within the scheme of these texts.^17^ It also raises interesting questions about the character of abstraction in mathematization in the precolonial Tamil country by situating it in social segregation and fragmentation, without losing sight of how the exercising of authority conditioned modes of abstraction and generalization through the particular occupational and social location of the different practitioners. These practices show how mathematical practice of the accountant was grounded in political economy of resource distribution and remained central to that social order.

The *Kaṇakkatikāram*’s section on land deals with mensuration involving land, where the presence of the measuring rod is constant.^18^ The person who performed the manual work of measuring, who belonged to a lower caste does not figure in the versified procedures of computation presented in these texts. The verses seem to address the accountant, who belonged to an upper caste and whose occupational responsibility involved working with measurements of land and its produce on a routine basis. The section on gold deals with persons assessing the purity of gold, which could be the goldsmith or a person trading in gold. Gold also assumes the form of money when it brings in peasants as share croppers and tax payers. There are problems where people such as wage workers ploughing the land, persons sharing profit in proportion to investment, salaried workers who received money from the treasury are present.^19^ In problems dealing with the conversion of paddy into rice, the cultivator and the merchant are constantly present, trying to exchange rice for money using the different units of capacity measures. Interestingly, the instance where one can imagine the presence of a woman worker in these texts is when a “rule of three” type of problem, the ratio of the number of times of pounding the rice is calculated in relation to the number of breaths taken by the worker.^20^ There are similar problems for the stone mason, for example, carving out pillars and grinding stones.^21^ There are verses dealing with the job of the water regulator, or the person in charge of irrigating land from the tank using different numbers of sluices and the time it takes to do so.^22^

The next section on fractions brings up problems of proportions, involving land and grain. The laborer and the trader figure prominently here: the agricultural laborer once more (except that here his wages are in fractional quantities and land is measured in fractions), creditors involved in land transactions, the laborer carrying grain, water mill operator, trader in oil, ghee and milk, people of two settlements betting on a fund, paying tribute, the money lender, the conch maker, the customs duty collector, the courier, the singer, the dancer, the person who assesses the productivity of land, and the livestock trader, and even the gambler.^23^ Such a variety of actors could be present in the computational ambience captured within the purview of a mathematical text shows the orientation of these practices towards measuring and counting in the everyday economic life of the seventeenth‐ and eighteenth‐century Tamil region. The carpenter making standard sized planks from timber and the weaver dyeing cloth of particular strengths do not appear in this particular manuscript, but they do appear, through the products they made, in the *Kaṇita Nūl*.^24^ Another interesting aspect of these texts is that the computations dealt with real world transactions in relation to land and its produce, which constitute the dominant concern; the world of the merchant or the trader is relatively less represented. The mathematics of the world of the merchant largely came under the rubric of “general problems” or what, as I have argued elsewhere, has been misleadingly labelled “recreational mathematics.”^25^

Eighteenth‐century Tamil society denied access to institutional education to the laboring caste groups and to women, with only the landed and the artisanal castes attending what was called the *tiṇṇai* school. But the *veṭṭiyāṉ*, who belonged to the untouchable and physically segregated caste was the person who physically measured land, handling the measuring rod in practice, as a worker in the contemporary revenue administration hierarchy. It is difficult to imagine him as a person unable to count, when he actually traced the boundaries of land with the rod. His immediate superior, the village accountant recorded what the *veṭṭiyāṉ* measured. The much‐contested threshing floor saw the worker mediating the distribution of grain, by measuring shares, much like the water regulator, the *nīrkkāraṉ* (sometimes called the *kampukaṭṭi*), whose job involved keeping time for moving waters for particular parcels of land, by constantly measuring and counting time with respect to area of land to be irrigated. All these workers would also have learnt their job from within their families and on the job, just like the accountant did. How could it be that the accountant was the only numerate agent in that society? What would constitute “numeracy” in such a context, especially when modes of learning were anchored in the mode of recollective memory based learning, where in writing became just yet another instance in the pedagogic process?^26^ Or still further, how might we understand the notion of apprenticeship, which concerns itself with transmission of knowledge in relation to only certain trades and professions, or particular kinds of work?

How did these subaltern life worlds shape contemporary knowledge production, whether that of the local practitioner or the hegemonic tradition of Sanskrit texts that primarily deal with the computational astronomical practices of upper caste Brahmins? Could context‐free knowledge emerge in a socially fractured society? To raise such a question is to propose that the history of knowledge in caste societies will have to reorient its central concerns towards the relationship, in knowledge production, between the mind and the hand in fundamental ways. This to me is the beginning premise for a social history of mathematics from below, in the context of early modern South Indian society.

## 3 The Accountant as the Practitioner

The *Kaṇakkatikāram* genre of texts clearly are the records of practices of the village accountant, which traverse aspects of both the pedagogic and the work place modes. In the early modern Tamil country, *kaṇakkuppiḷḷai*, or the accountant remained the master calculator, who as part of his profession, enumerated, assessed, and valued everyone's labor and produce. He represented the distant state authority in the local habitat. He recorded the activities of the settlement and provided a computational language to what everyone practiced. The institution and the persona of the *kaṇakkuppiḷḷai*—variously called accountant, scribe, or *karaṇam*—have attracted much historical attention in recent times. While one stream of scholarship demands of him a definite sense of history, as history was understood in the early modern period,^27^ the other characterizes him as the quintessential knowledge intermediary during the colonial encounter in the latter half of the eighteenth century.^28^ However, his most significant function was the actual work he carried out day to day influencing every aspect of village life. In the unfolding of the eternal tension between the local and the universal in historical terrain, the *Kaṇakkaṉ’s* literary prowess or his skill set has often been abstracted out of his political function in the local society, making him the powerful agent who sustained the status quo and subsisted out of it, through conscious ways of cultivating his skills in the mnemonic arts, traversing the world of letters and numbers. Bhavani Raman's *Document Raj* has brought out the social and the occupational world of the accountant in the early modern Tamil country.^29^ In her book, she explains how the accountant played a central role in the revenue management and record keeping aspects of village administration in the early modern Tamil speaking region from about the sixteenth‐century.

The accountant's primary job was to calculate and collect revenue of a village or a cluster, in cash or grain. In return, depending on the region, they were entitled to revenue shares or were patronized by the inhabitants of the locality. Local patronage meant constant monitoring of their occupation and abilities, which compelled them to keep up their proficiency and reputation. Scribes operated as agents of kings and influential households who tried to monopolize credit and fiscal networks, which made them very central to the expansion of fiscal domains into particular clusters in the eighteenth century. This enabled them to expand and consolidate their position in particular territories. Through their occupational function, we understand that the accountant decided on the amount of shares from the total produce of the village, routine assessment of land revenue, matters of inheritance and most importantly, was a witness of matters of local arbitrations. Using the locality accounts of the Chingleput area, which resulted from a survey during the years 1740–43, by Thomas Barnard, an official of the British East India Company to assess the revenue potential of a particular district of Chingleput in the Madras Presidency in South India. These surveys point to the village accountant's way of working and his proficiency in maintaining the accounts in terms of the contributions and entitlements of households and sections of laborers and the services rendered by them. Studying these locality accounts, the overwhelming presence of measuring as an activity of the different occupational groups in the village becomes evident, as much as the ways and means by which the accountant did his routine job.^30^ From the various types of registers that he maintained, according to the demands of the annual revenue assessment, we can see how his primary responsibility was to keep counting, measuring, and marking land and its produce, on the one hand, and collecting part of the produce as taxes and wages, on the other. Annually, he measured and marked the boundaries of the various types of village land and classified them in terms of boundaries and units in areas. He took stock of the land occupied by physical dwelling of the households, water bodies, temples, wasteland, grazing land, backyards, and public roads. He estimated the produce of different land parcels, assessed the actual production, divided the production in terms of the rights and entitlements of the inhabitants, allocated shares to the producers, to the temple, to the families of the service caste, including to himself, divided quantities of produce among shareholders, took parts of produce as taxes, and paid the laborers’ wages on the threshing floor. The act of measuring on the threshing floor—overseen by the accountant, but physically done by the *Veṭṭiyāṉ*—was a very contested and unhappy place, when the measurement was under constant scrutiny and contestation. The grain heap in the measuring vessel, or the arbitrary fixing of the value of the measures in terms of money, were not transparent processes of computation for the people involved but rather incited discontent and anger. The field deductions of shares on a routine basis kept the relation of proportions between units of measures very central to the accountant's job: a field of proportions that was pervaded with conceit and hatred, often for those who were receiving grains in assigned values.^31^

Here below, I will illustrate the nature of the accountant's job from the accounts of the year 1764, for one particular village, Thirupporur in the Chingleput district of the Madras Presidency, covered under the Barnard survey discussed above. The revenue was calculated separately for the revenue paying lands as payments in kind and for the revenue assigned lands, which are lands assigned to particular families or temples: for 65 measures of paddy, the produce was 848 *kalams*, 8 *marakkāl*, and 5 *paṭis* of paddy and likewise for all other grains produced.^32^ So, for the total of 81 and a half *kāṇi* of land, the total produce was 1160 *kalams*, 11 marakkāl, and 2 *paṭi* of all grains produced in that year. Then, the accountant began the business of deductions. First were amounts to be deducted from the revenue assigned land: For a produce of 10 *kalams*, 8 *marakkāls*, the wage workers received a share at the rate of 8 *marakkāls* per *kalam*. This was followed by deductions for the members of the service castes in terms of rate of grain measures per unit of land. Then the accountant calculated value of the grain in terms of money: for paddy at the rate of 3 *kalams*, 6 *marakkāl*s for one *varākaṉ* (unit of money), 269 *kalams*, 1 *marakkāl*, and 3 *paṭis* of paddy was 76 *varākaṉ*, 32 *paṇam*, and 50 *kāsu* and so on. The accountant then also collected tax from the village merchants. Then the final total revenue was calculated from which the share for the state and local public works allocated, and his signature attested in the register.^33^ This brief glimpse into the work of the accountant shows how he was a settlement's revenue accountant whose ‘skills of assessment, his ability to convert several disparate objects into a single value’ and his writing skill was ‘more than a functional mastery over techniques of storage; it was a skill connected to the recognition of patterns and calibration of assessment through the dexterous conversion of matter and value.’^34^ To perform these computations on a regular and routine basis, the accountant's training with the Tamil number tables at school and continuous practice on job, in the fields and at the threshing floor all came together to cultivate his facility with numbers. He also at times nurtured his skill through family‐based apprenticeship starting his career as an unpaid apprentice to relatives, where he worked hard to augment his skills in the recollective memory mode, until he became a proficient master of computation. However, we should not lose sight of the fact that his occupation and practice kept him the most active political figure, invested with the authority to measure, assess revenue, and authorize it on public books. As a political authority he aligned with the upper caste elite of the village settlement while maintaining registers of distribution of value to the labor of the different economic actors in the settlement.^35^ I would also like to point out that a long history of property relations looked through the prism of the accountant's computational practice is necessary. In our program, we are currently making an attempt to study these aspects in the medieval Tamil country using temple inscriptions and hope to make it available to students soon.

## 4 The Changing World of Computational Work in the Colonial Period

For the purpose of this discussion, I shall look at the changed role of the village accountant during his occupation's encounter with British colonialism through the East India Company during the late eighteenth and the nineteenth centuries in South India. In the early years of the nineteenth century, when legalized private property ownership in agrarian relations was introduced, we can witness a shift in the alignment between knowledge practices and production relations, traced through the office of the village accountant. This is one way how we can understand the changing character of numerate work and see how the realm of numbers and measures manifested the changes in the social relations of production.

Since the time of the large state formations in South India during the seventh‐century onwards, we have a huge corpus of historical records with details of production, taxation and distribution as activities of measurement and their records kept in the form of inscriptions.^36^ A long history of property is due for us to trace the instances of private property centered social relations and knowledge practices. Transactions involving the assessment of produce, credit entitlements, and commercial exchange were well established in South India from medieval times, if not earlier. Indeed, the expertise of the accountant‐practitioner's mathematics were well established by the seventeenth century. For now, confining ourselves to the East India Company's rule in the early nineteenth century, there was new system which put in place a legal and juridical machinery to legitimate private property in relation to the needs of revenue for the Company's political rule over an agrarian and mercantile world of production. The axis of the transmission of computational knowledge was redirected in the nineteenth century with the consolidation of the Company's rule, the establishment of property in land, and the range of demands this initiative imposed on social and economic terrains. The East India Company's revenue management rested much on securing private property in land through the regular maintenance of records, legible surveys, and easily convertible and accessible measurements.^37^ These interventions changed the topography in which numeracy operated. The establishment of land as private property brought with it concomitant changes to agricultural production and labor, and their counting and assessment. The revenue‐based entitlements and the labor quotient in assessing produce shares became two distinct entities in a different order of numbers, recorded in statistical registers tied to a regime of property rights.

The village revenue accountant continued to remain an important functionary to the new revenue organization. However, his computational expertise had to align with a new regime of field‐based assessment and measuring practices and the new dispensation was marked by a statistical orientation to record‐keeping. The number now had a new place in the political and social imaginary. It attested to the development of a new type of computational activity that was no longer the mode of proportional measurement, like in the case of the village accounts of Thirupporur presented above. It now had to assume an absolute value, dis‐embedded from the social relations of production. The changes of the accountant's work from doing proportional assessment as routine practice meant that the map of localized skills of numeracy also changed. Land now had to be measured not in the *Kaṇakkatikāram* style but with a different mode of mensuration, which brought together physical area with potential of productivity.^38^ It was not the measuring rod that the *Kaṇakkatikāram* made central to the procedures of computation any more. The acre became the new unit altering the practice of the accountants. The foot‐pole as the measuring rod was soon succeeded by the measuring chain with its links, and the processes of triangulation which contributed to the making of territorial scale in property, cementing the connection between measurement and state power.^39^

Such a new system of property and production assessment required a new kind of computational engagement. A different mode of work required institutions for new modes of training. Interestingly, the British East India Company tried out its new interventions in the pedagogy of mathematics not in the Tamil elementary schools it wanted to reform, but it did so by starting a new set of survey schools. One such school, started in 1804, for example was attached to the Madras Observatory and was supported by the Government. The purpose of this school was to train a new cadre of servants who could assist the Company's Engineers and Surveyors. The Company recruited its survey school students from the Anglo‐Indian orphanage of Madras city. These boys were considered ideal prospective surveyors and drafters—teachable and also removed from the social world distribution of revenue entitlements which characterized nature of the local political class.^40^ Also, such a cadre were meant to be independent of the occupational realm of the village accountant. Numerous occasions can be seen in the archival documents from this period which show how the local notables, in collaboration with the village accountants, tried to obstruct the surveying work carried out by the new cadre. This meant that a mere training and the making of expertise in measurement was not sufficient. The colonial state needed more than disinterested knowledge and statistical and surveying instruments. They had to enlist the support of local skilled personnel to quantify and classify productive resources. There were multiple layers of recording numbers. The information compiled locally in palm leaves were transcribed into statistical tables in English, which then travelled out of the settlement into the district revenue offices of the Company, en route to the Presidency capital where it was scrutinized through the lens of a political economy of revenue extraction, before it embarked on a long journey to the metropolis.

Such traffic in records created a large revenue establishment comprising of measurers, calculators, and scribes. The accountant's computational practices had to be incorporated into this new revenue system, making them the new artefacts of colonial rule. In this process, the significance of number changed for the local public. Assessment of land and produce was no longer confined to the threshing floor and the locality. It had to be settled at an office far from the locality. This meant that the higher revenue officers of the Company were always suspect of the village officers. Not just them, but the cultivators and the measuring public of the locality also were wary of the new kind of revenue administration. Previously, land related computations might have been an expert practice, but then the methods and norms of measurement and computations were familiar to the people. But now, calculation of the physical area of land, units of measurement to assess its produce and the tax calculations were all in profusely numerical registers, neither transparent nor accessible to the people of the settlement. This meant that a new and additional layer of interpretation of these numbers, and the people who could do the job of this interpretation became the new mediators between the Company state and the people, creating a new form of dependency on these mediatory interpreters. The cultivators on the other hand could not any longer understand the methods of the accountant, who has now become the powerful local agent of the new colonial dispensation.^41^

As far as the local map of required skills were concerned, apart from the computational skills of the accountant's job, other skills such as in record keeping, translation and surveying became the new requirements. There were offices at various levels from the village to the district Collectorate, which created a new job market involving a variety of measuring and writing expertise. These jobs were precarious because it seemed to be a time of rapid hire and fire. However, what is of significance to our discussion here is that the *Kaṇakkatikāram*’s ways of problem‐solving had to confront the new requirements of calculation in a new administration. The accountant‐practitioner however still cannot be displaced as his skill base remained indispensable to the Company state through the first half of the nineteenth century. But he was also suspect at the same time, and this lack of trust induced a set of reforms on the moral plane, which were to be addressed by educational reforms which the Company introduced since the 1820s.^42^

The story of how the actual need for skills by the colonial state had to orchestrate its rhetoric of public education, importantly involved a changing topography of numeracy. The regime of these new numbers pervaded the household, the market, the school, and the offices of the revenue administration. In this changing landscape of numeracy, we see how the rule of private property and production had shaped the way computational practices, both in the school and at the work place, to render them commensurate with the rule of extraction and exploitation by the British East India Company.^43^

## 5 The Public and Computational Work of the Accountant

We can see the clear professional and political role of the occupation of the accountant both during the precolonial period and during the rule of the Company. In attempting a social history of mathematical practices through the lens of the occupational practices, I argued that modes of the computational work of the practitioner cannot be divested from the politics of the profession itself. Even in this case, the practitioner's records need to be retrieved and the world of his practice has to be reconstructed, but with care taken not to privilege that mode of practice in such a way that it would constitute yet another hegemonic tradition in the history of mathematics. One possible way to prevent this from happening is to simultaneously retrieve the lives of the public whom the accountant worked for and worked with. In the case of the village accountant, the historian Sivasubramanian has collected certain oral histories where he documents from among the people and their memories certain virtues as well as vicious qualities of the accountant.^44^ As mentioned earlier in this paper, it is very common among the Tamil‐speaking public to identify the act of computation with the act of manipulation in common parlance. Central to this perception is also the dislike incited by the accountant's role in aiding his master, the administration or the rich landlord, the Zamindar, a common theme frequently appearing in popular culture. In this collection of cultural lore, Sivasubramanian documents in detail the perception of the accountant from literary sources of the precolonial era as well as from oral histories that speak of the accountant and his practices, which tend not to celebrate his virtues but rather to talk about the malpractices and manipulations of the occupation. There were certain material conditions under which these perceptions arose. One common manipulation that the accountant engaged in was to occupy the commons around the canals and cultivate those lands himself, while reclassifying them as uncultivated dry land in the register. The accountants also often exploited the labor of the oppressed caste groups and the peasantry by demanding that they work free on the accountant's own lands. If anyone dare to refuse to perform free labor for the accountant, it was almost certain that their land and plough would be instantly “cursed to haunt their lives.” Hence, one of the pioneers of anti‐caste movements in the Tamil country, Ayotheedas Pandithar, argued to the colonial government that the way to ensure an end to these exploitative practices of the accountant would be to ensure that no accountant owns land of his own.^45^

The author has also collected proverbs and folklore concerning the accountant. Consider the rather harsh usage that Sivasubramanian records from the people: ‘Kaṇakkaṉ cettāl piṇam, Kaṇakkaṉ āttāl cettāl maṇam,’ which means that if the *Kaṇakkaṉ* dies, it is just a matter of a corpse, but if his mother dies, then it is an occasion. If the *Kaṇakkaṉ* dies, people are relieved that it is just a matter of burial, and they get it done with, but if his mother dies, then everyone has to be present so that they do not upset him, making it a compulsory occasion, often involving monetary expenditure.^46^ This sentiment resonates with another adage Sivasubramanian records that says if the *Kaṇakkaṉ* is upset with you, your land will also be upset. That is to say it is up to the *Kaṇakkaṉ* to define the boundary of your land and if you upset him, he will mess about with your land. If anyone upsets the accountant, he could be sure, in fact, that he would lose his own land. Also, the very act of measuring using chains and rods was considered manipulative too. It was widely believed that the accountant would tighten the chain at exactly the moment when it matters in order to manipulate the measure. It was not the case that the superior revenue officials were in a position to verify and check these practices of the accountant. Another proverb mentions that the knot tied by the accountant cannot be removed even by the District Collector.^47^ In his account, the historian has also documented how in certain street games played by children, the figure of the village accountant occurs as one to be vilified and ridiculed, such as in one game in which the cloth figure of the accountant would be thrown on the ground to be spat upon by all players of the game.^48^ This perception of the accountant was not unique to the Tamil‐speaking region alone. This seems to have been widespread across the subcontinent. For example, in the Telugu‐speaking region of South India, Edgar Thurston talks about how the accountant was detested by the people, where he lists certain proverbs such as the following: “Though babies are sold for a pie each, we do not want a Kanakka baby” and “Wherever you meet with a Kanakka child or with a crow's young one, put out its eyes.”^49^ Such violence in the perceptions evident in such popular lore were not isolated instances in the past.

For another example, there is one particular story that speaks of the power of the accountant and the hatred he managed to nurture among the people. A village accountant wanted to take revenge on the people who detested him. He did not want to give them the joy they would feel once he died. So, he gathered all the people of the village at his deathbed and told them that he had indeed caused enormous hardship to them and his soul would not rest in peace until he would be punished by all of them. Therefore, he requested them not to carry his dead body to cremation with fanfare as is normally expected but for it to be dragged along the streets where it would be beaten by each and every person of the village all the way to the burial ground. The villagers were taken by surprise even if many nurtured a secret joy at the possibility of being able to deliver such a punishment. When the accountant did pass away, the villagers respected his death wish and dragged the corpse, taking turns beating it with sticks. But when they reached the burial ground, much to their surprise, the police encircled them and arrested them on the charge of murdering the accountant. Thus, the accountant managed to exact his revenge on the people, who detested him.^50^ The cunning mind of the accountant and the hatred he incited among the people due to his profession, premised on his computational proficiency is starkly revealed in this story.

Such stories and many other popular lore about the village accountant point to how the computational proficiency of his job merged with the manipulative nature of his occupation, to an extent that the Tamil word for computation or mathematics, *Kaṇakku*, has come to assume the meaning of cunning and manipulation. A long history of the occupation of the accountant is necessary to unravel this relation between mathematical practices and its consequences for the people. This is the subject of ongoing work in our research program on the social history of mathematical practices through which we hope to show how the pursuit of mathematics was political, when studied through occupations and learning practices in history.

## 6 Concluding Remarks

In the discussion above, I have explained the nature and orientation of the occupational practice of the accountant as the mathematical practitioner. While the orientation of the computational work is political, is a job, is an occupation, we also find in the texts discussed a quest for generalization, going beyond the practical. This could be seen as emerging out of pedagogic and work practices, realms in which the profession of the accountant was made and sustained. If so, how do we recognize these as different modes and styles of abstraction, grounded in practice, so that we do not celebrate abstraction outside their historical context, which contributes to hegemonic models of knowledge, in turn subverting possibilities of democratic knowledge making processes? Could styles of this kind become part of national and civilizational histories? In such practices, practical mensuration and the computational process were in intimate relationship, couched within each other, when methods such as the rule of arithmetical proportions were deployed and mastered. But they also involved the political necessity of a revenue administration, rendering the rule of proportion a political device. The mode was one of practical mathematics as is often characterized in the history of mathematics, but a mode of practice made mathematical through acts of mensuration. To demand from this practitioner a mode of contemplation, to ask of him the generalized properties of the circle or the square might be misleading just because this was not part of his orientation. The constant search for generalization to be classified into categories such as the pure and the applied has often caused the practitioner's knowledge to be seen as folk, that is, inadequately mathematical or not theoretical enough. The world of mensuration was not any less valid than the pursuit of disembodied knowledge just because its orientation was not the quest for a geometrical blueprint of the world.^51^

If mathematical practices are to be understood as work that evolves into processes emerging out of politically directed institutions, irrespective of the styles of abstraction and argumentation, might we then ask if there were mathematical practices to be found in history that did not necessarily lend themselves to processes of alienation? Especially in socially fragmented contexts, when children could not come together to learn the world of proportions, beyond the confines of their caste, what would be universal basis for that knowledge, is a question that would provide us with insights to democratize the same knowledge. Such a project then will have to draw upon other valuable aspects from similar histories, when mathematics also provided children, spaces to learn it with joy and shaped their experience with mathematics differently. In the Oriya‐speaking region of India for example, there is a corpus of texts in mathematics which testify to a pedagogic culture of learning mathematics as musical compositions and performance. Such traditions also need to be studied by the social historian, even if they were an integral part of the same contexts of social fragmentation.^52^

## Footnotes

n1 Senthil Babu 2022, chapter IV, on 182–214.

n2 See https://ifpindia.org/projects/history‐of‐vernacular‐mathematical‐practices‐in‐india/. Also my book, Senthil Babu 2022, is an attempt in this direction.

n3 Robson 2009a, on 273.

n4 Robson 2009b.

n5
*Kaṇakkatikāram* 1783. This manuscript is edited and available as *Suriyapūpaṉ Kaṇakkatikāram (Pakuti II)* 2007. But in the essay, we shall refer to this as *Kaṇakkatikāram* 1783.

n6 Mahadevan 1978.

n7 Annamalai 1988.

n9 Both these manuscripts are in the collections in the Government Oriental Manuscripts Library, Chennai, the Thanjavur Tamil University Manuscripts Library, and in the Sarasvathi Mahal Library in Thanjavur. As part of the research program, we are creating an online digital public archive of all these available manuscripts.

n8 I draw upon my book in this section to set a context and describe the nature of the historical material available for a history from below, that is, a different history of mathematics in South India. The following sections with description of the *Eṇcuvaṭi* and the *Kaṇakkatikāram* and the subsequent section on the nature of the everyday job of the accountant are drawn from my book, Senthil Babu 2022. For historical discussion of the profession of the accountant. See also Raman 2012.

n10 These manuscripts, interestingly, were also printed and published through the nineteenth century. As palm‐leaf manuscripts, they are found in the library repositories mentioned above. However, *Kaṇakkatikāram* manuscripts are few in numbers when compared to the *Eṇcuvaṭi*. Both the available palm‐leaf and the print versions will be available from our digital archive which will be notified through our project site, https://ifpindia.org/projects/history‐of‐vernacular‐mathematical‐practices‐in‐india/.

n11 The direct evidence of the presence of these schools are recorded in the British colonial surveys undertaken during the early nineteenth century which point to their existence at least from the eighteenth‐century, if not earlier. For a discussion about these schools in detail, see Senthil Babu 2022, on 122–137.

n12 Senthil Babu 2022, chapter III, section on the “Learning of Mathematics,” on 142–152.

n13 Senthil Babu 2022, chapter III, section on “Tiṇṇai Curriculum, Pedagogy and Caste,” on 169–173. See also Senthil Babu 2007.

n14 Senthil Babu 2022, on 163–169.

n15 Sathyabhama and Subramaniam 2007; Sathyabhama 1998.

n16 As part of our research program, we have found that these texts were published in the Madras Presidency through the nineteenth century, almost once in every two years. This points to their continuous use and circulation. The information about the palm‐leaf manuscripts available in various libraries are mentioned already. Along with the printed editions, information about various versions and copies of these texts in different locations will be collated and available for open access in the archive we are developing.

n17
*Kaṇakkatikāram* 1783.

n18 Ibid., on 119–162.

n19 Ibid., on 164–225.

n20 Ibid., on 226–252.

n21 Ibid., on 253–264.

n22 Ibid., on 264–271.

n23 Ibid., on 271–336.

n24
*Kaṇita Nūl* 1999.

n25 Senthil Babu 2022, on 71–80.

n26 I discuss recollective memory based learning in the pedagogic processes of south Indian society in the precolonial period in chapter 3 of my book more elaborately: Senthil Babu 2022, on 159–160. See also Carruthers 1990.

n27 Narayana Rao et al. 2001.

n28 Wagoner 2003. See also Bayly 1999.

n29 Raman 2012. We draw upon her work in this section to describe the social and occupational profile of the *Kaṇakkaṉ* in the pre‐colonial Tamil region.

n30 Srinivas et al. 2001. See also the unpublished doctoral dissertation of Pushkala who has reconstructed the accounts for a different set of localities using the same corpus: Pushkala 1997.

n31 Senthil Babu 2022, on 88–89.

n32 These are capacity measures in the Tamil system, which are ordered in particular ratios, such as 12 *marakkāls* will make one *kalam*, which is the highest unit. And *varākaṉ*, *paṇam*, and *kāsu* are units of money.

n33 Srinivas et al. 2001.

n34 Raman 2012, on 61.

n35 It is interesting that in the literature on scribal practices in the history of mathematics, the computational virtues of the accountant are often discussed in tandem with the kind of problems his job and training would involve, and only rarely with the political implications of his practice, which is appropriately termed “numerate justice” in Robson 2009b, on 419, in the case of old Babylonian scribes.

n36 Subbarayulu 2011; Karashima 1984.

n37 This is discussed elaborately in Raman 2012.

n38 The changed system of revenue administration is studied in detail by Mukherjee 1962.

n39 For a detailed explanation of these processes during the colonial transition, please see Senthil Babu 2022, chapter IV. For an account of the changing ways of surveying and mapping in British India, and more specifically on the revenue survey and new pedagogic initiatives in this period, see Edney 1997, on 170–175.

n40 A recent essay discusses in detail the relationship between the school attached to the observatory and the early surveying practices. See Prashant Kumar 2022.

n41 The reshaping of computational knowledge in this period is discussed in detail in my book, Senthil Babu 2022, on 177–182.

n42 One of the important architects of these reforms was Thomas Munro, who was then the Governor General of the Madras Presidency who did an elaborate and detailed survey of indigenous schools in the Presidency which became consequential for all educational interventions through the rest of the nineteenth century in the Madras Presidency. The archival records of these surveys are reproduced in Dharampal 1983.

n43 The details of this story is discussed in detail in Senthil Babu 2022, chapter IV, on 195–214.

n44 Sivasubramanian 2002, on 82–112.

n45 Sivasubramanian 2002, on 98. Ayotheedas Pandithar's intellectual contributions in forging a vibrant critique of caste society is well recognized now in Tamil political discourse. The publication of his collected writings have become an important source for political movements of the oppressed people. See Aloysius 1999.

n46 Sivasubramanian 2002, on 99.

n47 Ibid., on 99.

n48 Ibid., on 101.

n49 Thurston 1909.

n50 Sivasubramanian 2002, on 103–106.

n51 Bennett 1998.

n52 A *Descriptive Catalogue of Oriya Mathematical Manuscripts in the Government Museum of Orissa* is under preparation by Gauranga Dash and will be published shortly.
